# The Direct Anterior Approach to Primary Total Hip Replacement: Radiological Analysis in Comparison to Other Approaches

**DOI:** 10.3390/jcm10112246

**Published:** 2021-05-21

**Authors:** Artur Stolarczyk, Magda Stolarczyk, Piotr Stępiński, Monika K. Dorocińska, Maciej Świercz, Jakub Szymczak, Krystian Żarnovsky, Albert Żuchniewicz, Bartosz M. Maciąg

**Affiliations:** 1 Department of Orthopaedics and Rehabilitation, Medical University of Warsaw, 02-091 Warszawa, Poland; drstolarczyk@gmail.com (A.S.); Piotr.stepinski01@gmail.com (P.S.); dorocinskamonika@gmail.com (M.K.D.); maciek.swiercz2@gmail.com (M.Ś.); jakubszymczak92@gmail.com (J.S.); k.zarnovsky4@gmail.com (K.Ż.); albert1152@gmail.com (A.Ż.); 2 3rd Clinic of Internal Medicine and Cardiology, Medical University of Warsaw, 02-091 Warszawa, Poland; ortopedia@mssw.pl

**Keywords:** THR, approach, hip, anterior

## Abstract

Total hip arthroplasty (THA) is currently considered the most effective treatment for end-stage hip osteoarthritis (OA). The surgery can be performed via a number of different approaches, including direct anterior (DAA; Smith–Petersen; Hueter), anterolateral (ALA; Watson–Jones), direct lateral (LA; Bauer), posterior (PA; Kocher–Langenbeck), and posterolateral (PLA). There is still a dispute over the optimal technique. The aim of this systematic review was to assess how different surgical approaches toward a THA influence the prosthesis elements’ positioning. We conducted a literature search of Scopus, ScienceDirect, PubMed, Embase, and The Cochrane Library. We evaluated studies in terms of the first author’s name, country, publication year, type of surgical approach being compared to the direct anterior approach, any significant differences at baseline, sample size, and radiographic analysis. A subanalysis of each approach in comparison to the DAA revealed differences in terms of all analyzed implant positioning radiographic parameters. There is still an insufficient number of randomized controlled studies that include radiological analyses comparing THRs (total hip replacements) performed using DAA with other approaches. Implant placement is a crucial step during a THR and surgeons must be aware that the approach they use might impact their judgment on angles and spaces inside the joint and thus alter the implant positioning.

## 1. Introduction

Total hip arthroplasty is currently considered the most effective treatment for end-stage hip osteoarthritis (OA) [1]. However, there is a continuous dispute over selecting the optimal technique [2,3,4], with the most popular being direct anterior, anterolateral, direct lateral, posterior, and posterolateral. The choice of approach determines which tissues, including muscles and tendons, need to be dissected in order to reach the joint, which structures should be avoided, and how difficult it is for a surgeon to correctly position the implants [5,6]. A direct anterior approach (DAA; Smith–Petersen; Hueter) is considered the least traumatic as it utilizes the intermuscular plane between the sartorius, rectus femoris, and tensor fasciae latae muscles, with no need for the dissection of any of them. Even though it was introduced many years ago [7], it is currently gaining in popularity, along with the general tendency toward minimally invasive surgery [8,9]. The key to its recognition are postulated positive effects on prosthesis stability and patient satisfaction. There are several claimed advantages of DAA when compared to some other approaches, including faster rehabilitation and reduced postoperative pain [10,11,12].

However, significant differences in clinical (functional) outcomes are usually observed for only a few months postoperation and we have found no sufficient scientific evidence of DAA’s long-term superiority [10,11,12]. Inevitably, there are also claimed downsides of this approach, such as a flat learning curve, an associated increase in the rate of complications, and worse functional outcomes when compared to other approaches [10,13,14].

Even though the relative effects of the direct anterior approach have already been covered by several systematic reviews and/or meta-analyses, we have found that none of them included radiographic assessment of prosthesis placements [10,13,14]. Furthermore, while there are no definitive conclusions on the choice of the approach, such a review could play a significant role in the discussion, addressing a common allegation that the DAA learning curve might often have an impact on proper implant alignment. Radiographic prosthesis position evaluation is essentially based on acetabular cup anteversion and inclination. The most notable application of these parameters is attributed to finding the safe zone introduced by Lewinnek et al. [15] to predict which positions promote dislocations. Although the values and the evidence they used were repeatedly contested, the safe zone remains an important guideline for prosthesis placement.

The objective of our study was to collect and review the available data regarding radiographic assessments of prosthesis placement after total hip arthroplasties performed using the direct anterior approach compared to other common approaches.

## 2. Materials and Methods

**Figure 1:**
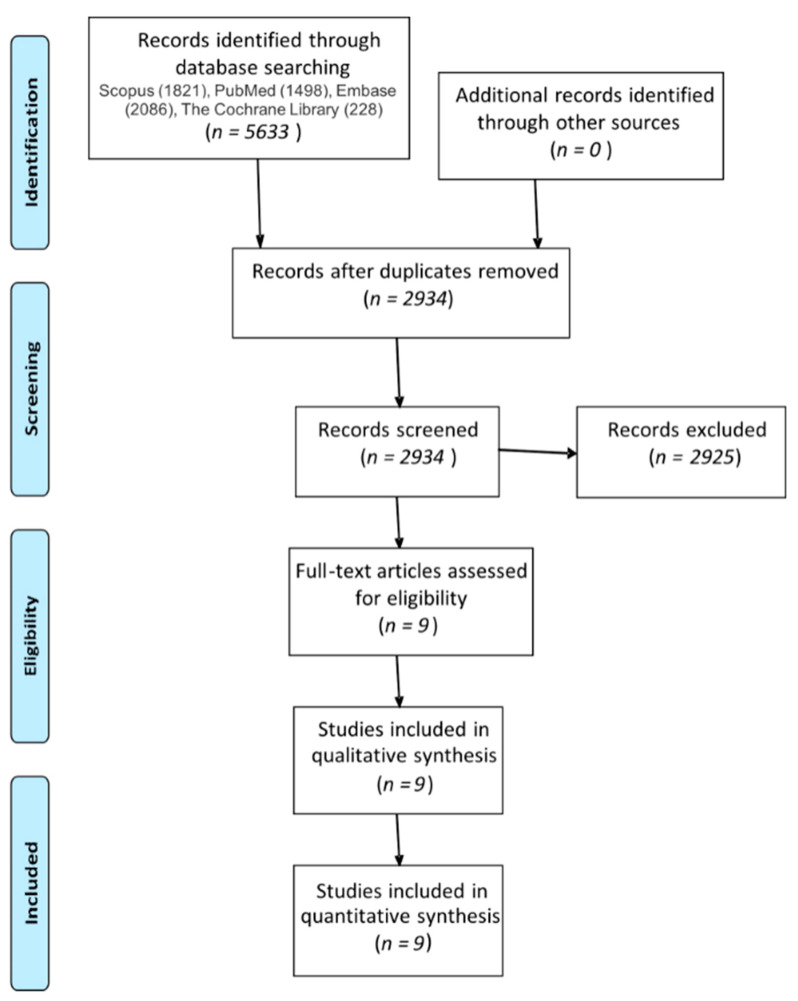
PRISMA flow diagram.

This study is reported in accordance with the Preferred Reporting Items for Systematic Reviews and Meta-Analysis (PRISMA) statement [16] (Figure 1) and the Cochrane Handbook for Systematic Reviews of Interventions [17]. This study protocol was registered in the International prospective register protocol of systematic reviews PROSPERO (PROSPERO number CRD42019122675).

No institutional review board approval was required for this review because the study included data that had been published previously.

We conducted an English language literature search of Scopus, ScienceDirect, PubMed, Embase, and The Cochrane Library in January 2021 without restriction in terms of the date. The following search terms were used: ‘total hip replacement,’ ‘total hip arthroplasty,’ ‘THA,’ ‘THR,’ ‘anterior,’ ‘direct anterior,’ ’anterior supine intermuscular,’ ‘Hueter approach,’ and ‘Smith–Petersen.’ Search terms were combined using the Boolean operators ‘AND’ and ‘OR’ in accordance with the methodology used by Yue et al. [18].

In this review, the inclusion criteria consisted of randomized clinical trials involving patients over 18 years old (with primary hip osteoarthritis that was treated surgically), studies comparing the direct anterior approach (DAA) with other approaches, and consisting of radiological analysis. We excluded non-English studies, studies for which only abstracts were available, review or non-comparative studies, and research in which bilateral hip replacement or hemiarthroplasty surgeries were analyzed.

Three independent researchers (B.M., K.Ż., M.D.) evaluated the final set of studies in terms of: the first author’s name, country, publication year, type of surgical approach being compared to the direct anterior approach, any significant differences at baseline, sample size, and radiographic analysis (femoral stem alignment, mean radiographic cup inclination, mean radiographic cup anteversion, mean radiographic cup abduction, position in Lewinnek’s safe zone).

We provide a narrative synthesis of the findings from the included studies, which was structured around the type of intervention, the targeted population characteristics, the outcome, and the intervention content. The primary outcomes were the femoral stem alignment, mean radiographic cup inclination, mean radiographic cup anteversion, and mean radiographic cup abduction. Secondary outcomes were the leg length following the surgery and the position in Lewinnek’s safe zone. Furthermore, subgroup analysis regarding the type of approach toward the THR was performed. We considered studies comparing DAA to one of the following: LA (lateral approach; Bauer), PA (posterior approach; Kocher–Langenbeck), or PLA (posterolateral approach). To provide a structured summary of the questions asked in this narrative review, a table depicting the study characteristics in accordance with the PICOS strategy was prepared (Table 1).

### Assessment of Methodological Quality

The Cochrane Collaboration’s tool [19] for assessing the risk of bias was used. Any disagreements between them over the eligibility of particular studies were resolved via discussion with a fourth reviewer (A.S.). Cohen’s kappa coefficient [20] was calculated for the interrater agreement between reviewers following the assessment of the studies’ eligibility. Kappa values ≤ 0 were interpreted as indicating no agreement, 0.01–0.20 as none to slight, 0.21–0.40 as fair, 0.41–0.60 as moderate, 0.61–0.80 as substantial, and 0.81–1.00 as almost perfect agreement.

## 3. Results

### 3.1. Literature Selection

A narrative investigation of PubMed, Embase, Scopus, and Cochrane Collaboration of Systematic Reviews produced 5633 potentially eligible studies. We used EndNote X9 to remove duplicated studies (2699). The remaining 2934 studies were then read by two independent researchers. A total of 2925 studies were excluded due to not matching the selection criteria. In the end, nine studies were included in the review [8,21,22,23,24,25,26,27,28].

#### 3.1.1. Study Characteristics

Nine randomized controlled trials (RCTs) were analyzed in this systematic review [8,23,24,25,26,27,28,29,30]. Four of them compared DAA to the standard lateral approach (LA) [8,21,22,27], three of them compared DAA to the standard posterior approach (PA) [19,24,28], and two compared DAA to the posterolateral approach (PLA) [25,26].

#### 3.1.2. Participants

Altogether, the radiological analysis of the implants’ positioning was performed in 994 extremities: 500 in DAA vs. LA, 287 in DAA vs. PA, and 207 in DAA vs. PLA (Table 2). The age and gender of participants, inclusion/exclusion criteria, and implants used for the THRs are presented in Table 2.

#### 3.1.3. Risk of Bias within Studies

The risk of bias within studies was assessed with the applicable part of the Cochrane Collaboration’s tool for assessing the risk of bias, as described in the Materials and Methods section. The results of this assessment are presented in Table 3.

### 3.2. Femoral Stem Alignment

**Figure 2:**
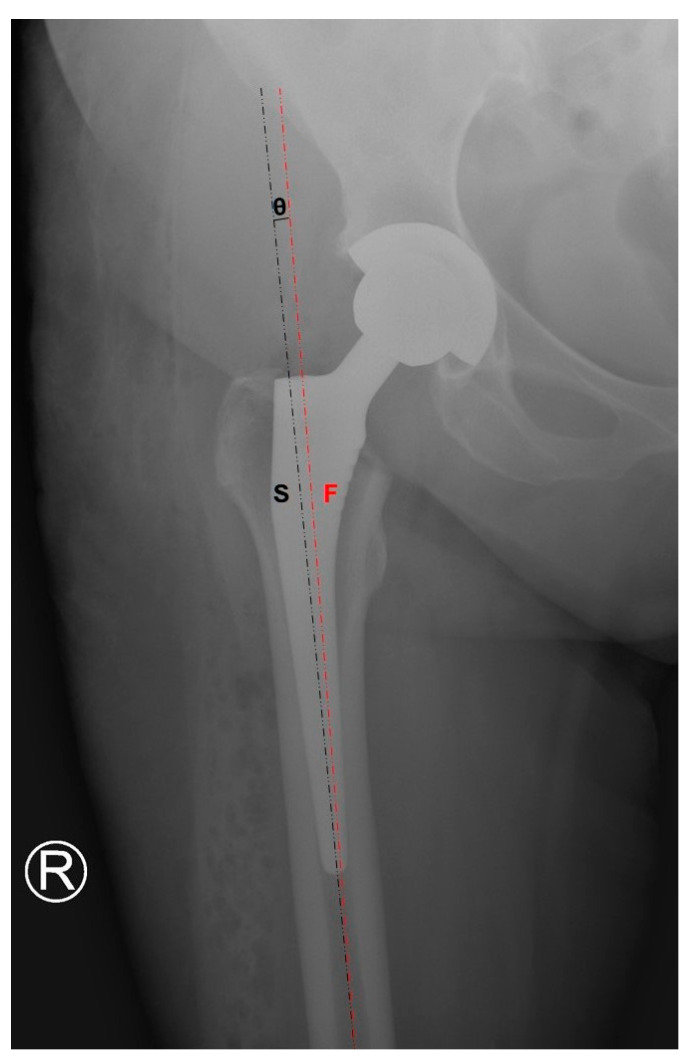
Femoral stem alignment measurement.

Seven RCTs compared the femoral stem alignment (Figure 2) in patients that were operated on using DAA to other approaches [8,21,22,23,25,27,28]. Four of them analyzed groups that were operated using LA, two with PA, and one with PLA (Table 4). The median femoral stem angle was 1.23° varus in DAA, and 2.37° varus in two studies that analyzed LA. However, the reason for such a significant difference might have been a difference in the number of participants in the comparative group in Dienstknecht et al.’s research (55 vs. 88). In Brun et al.’s work, there was no difference in the mean femoral stem position between the two trial groups (*p* = 0.443). In Cheng et al.’s work, the mean femoral stem orientation was −1.60° varus. In Reichert et al.’s work, the alignment of the femoral stem was not measured in value, but the researchers provided information stating that the stems in the DAA group were assessed to be in a neutral position in 92.5% of cases, varus in 5.5%, and valgus in 2%, while in the LA group, 94% were positioned neutrally, 4% varus, and 2% valgus. In Taunton et al.’s work, the researchers remarked that in the DAA group, there were four stems in varus, while in the PA group, there were six stems in varus and two in valgus.

### 3.3. Mean Radiographic Cup Inclination

**Figure 3:**
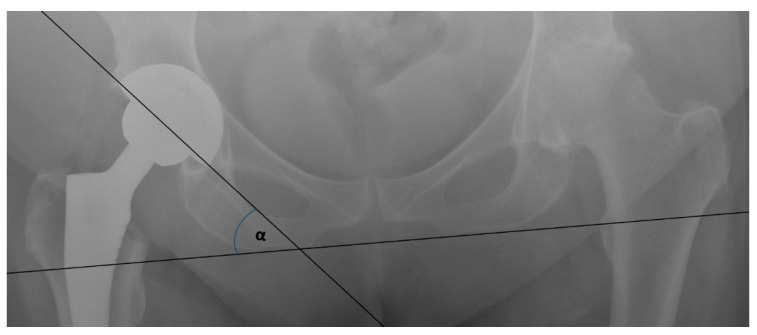
Cup inclination measurement.

Nine RCTs compared the mean radiographic cup inclination (Figure 3) between patients that were operated on using DAA and patients that were operated on using other approaches [8,21,22,23,24,25,26,27,28]. Four of them analyzed the groups that were operated on using LA, three using PA, and two using PLA (Table 5). The most significant difference was observed in the DAA vs. LA subanalysis. In the analysis of these two RCTs, the mean cup inclination angles in the DAA and LA groups were 42.68° and 46.29°, respectively. Such a difference might have been due to the unequal number of participants in both included studies (73 vs. 50 and 55 vs. 88). However, in Brun et al.’s work, where the number of patients in both trial groups (80 vs. 84) was comparable, the degree of cup inclination was significantly higher in the DAA group than in the LA group (mean difference = 2.5°; *p* = 0.023). A similar observation was made in Nistor et al.’s study, where the cup inclination angle difference between the DAA and LA groups was statistically significant with a *p*-value < 0.001, with lower values in the DAA group. Three RCTs measured the cup inclination angle in the DAA and PA groups, but no statistically significant difference in those studies was observed. Meanwhile, two RCTs compared this angle between DAA and PLA. The mean radiographic cup inclinations were measured to be 43.14° and 42.05°, respectively.

### 3.4. Mean Radiographic Cup Anteversion

**Figure 4:**
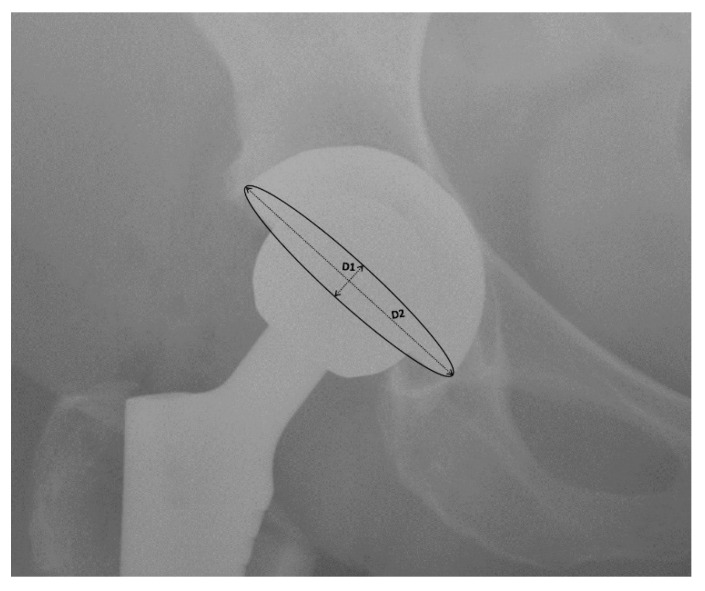
Cup anteversion measurement.

Six RCTs compared the radiographic cup anteversion (Figure 4) in patients that were operated on using DAA to those patients operated on using other approaches [23,24,25,26,27,28]. Three of the RCTs analyzed groups operated on using PA, two using PLA, and one using LA (Table 6). The mean radiographic cup anteversion in the DAA group was 21.42° compared to 23.01° for the other approaches. However, a subanalysis of DAA vs. PA and DAA vs. PLA showed more significant differences in these measurements. Comparing DAA with PA, it was found that the mean radiographic cup anteversions were 26.52° and 22.70°, respectively. Moreover, in an analysis of the DAA vs. PLA subgroup, the angles were 18.35° vs. 23.20°, respectively. Furthermore, the angle of the cup anteversion was significantly higher in the DAA group compared to the LA group (mean difference = 3.6°; *p* < 0.0001). In four of the mentioned RCTs, the difference in cup positioning was statistically significant with a *p*-value < 0.05. Moreover, in Cheng et al.’s work, this difference was also recognized and was nearly statistically significant (*p* = 0.06).

### 3.5. Leg Length

Three RCTs reported an analysis of leg length discrepancies following THRs [21,24,28]. However, different methods of reporting these values were used. Reichert et al. reported two cases (3%) with a discrepancy of more than 1 cm in comparison to the non-operated limb in the DAA group and 3 cm in the LA group (6%). These values are difficult to compare due to the quite significant difference in the numbers of participants in these studies. Taunton et al. measured the median leg discrepancy (2 mm in the DAA group and 3 mm in the PA group). The result was found to be statistically non-significant (*p* = 0.222). Furthermore, in Brun et al.’s study, there was no difference in the mean leg length between the DAA group and LA group (*p* = 0.164).

### 3.6. DAA vs. LA

In all four RCTs comparing DAA to LA, the femoral stem positioning did not differ significantly between the groups. In Brun et al.’s study, the angles of both the cup inclination and anteversion were significantly higher in the DA group when compared to the DLA group (mean difference = 2.5°, *p* = 0.023 and mean difference = 3.6°, *p* < 0.0001, respectively). Moreover, in Nistor et al.’s study, the difference in cup inclination angle between the DAA and LA groups was statistically significant with a *p*-value < 0.001, with lower values in the DAA group. In two other RCTs, the difference in cup inclination angle was not found to be statistically significant. However, there was a significant difference between both studies in terms of the value of this angle. In Reichert et al.’s study, the mean value of this angle in both the DAA and LA groups was almost 10° lower than in Dienstknecht et al.’s research: 38.6 ± 5.7° vs. 48.1 ± 6.0° and 40.3 ± 6.2° vs. 49.7 ± 6.0°, respectively. Such differences might have been caused by the unequal number of participants between both compared groups. In Dienstknecht et al.’s research, the DAA group consisted of 55 participants, while the LA group had 88 participants. In Reichert et al.’s study, the DAA group consisted of 73 patients, while the LA group had 50 patients. In this particular study, such a difference between the groups might have been due to the loss to follow-up of 21 patients from the LA group (29.6%), mainly due to the ‘lack of time and interest’ (22.5%).

### 3.7. DAA vs. PA

With regard to the femoral stem alignment and cup inclination, three RCTs reported minor changes that were not statistically significant. However, in Taunton et al.’s research from 2014, a statistically significant difference was observed with regard to cup anteversion, with higher values in the DAA group (*p* = 0.004). In Cheng et al.’s research, similar observations were made with nearly statistical significance (*p* = 0.06). On the other hand, in Taunton et al.’s study from 2018, there was no disparity in terms of the cup anteversion in any group of patients (*p* = 0.21).

### 3.8. DAA vs. PLA

In both RCTs comparing DAA to PLA, no information concerning leg length after the THR femoral stem alignment or cup abduction angle was reported. Both studies provided statistically significant results of the measurement of cup anteversion angle (*p* = 0.0005 and *p* = 0.02), with lower values for DAA in comparison to PLA. However, values of this angle in both groups significantly differed between studies. In Barret et al.’s study, this angle was 20.1 ± 5.9° and 25.8 ± 8.1° for DAA and PLA, respectively. In Zhao et al.’s study, these values were 17.1 ± 2.1° and 21.3 ± 2.4°, respectively. These two RCTs provided different conclusions regarding the measurement of the cup inclination angle. In Barret et al.’s work, the authors showed that this angle was statistically higher in patients that were operated on using DAA than in the PLA group. These observations were not confirmed by Zhao et al.’s research in which these values were lower in the DAA than in the PLA group, but this difference was not statistically significant (*p* = 0.57). Such differences may be explained by the higher BMI values in Barret et al.’s research (29.1 ± 5.0 kg/m^2^ vs. 24.35 ± 3.10 kg/m^2^, respectively) and the fact that in Zhao et al.’s research, the inclusion criteria contained hips with residual dysplasia (Crowe I and II: DDA group = 6, PLA group = 7) and patients with femoral neck necrosis (Ficat III or IV: DDA group = 13, PLA group = 13). Hips with such characteristics were not included in Barret et al.’s research.

## 4. Discussion

Achieving the perfect stem and cup position during a THA is one of the toughest challenges. It is estimated that positioning the hip rotation center in 40° of inclination and 20° of anteversion will allow for a good clinical outcome [29,30]. Defining the optimal cup position is one thing, but achieving the targeted position in a reproducible way is even more difficult [31,32].

Proper cup orientation is believed to be the key factor toward achieving a proper cup-head contact area and minimizing its wear [33,34,35,36]. There is also an ongoing debate about whether the so-called “safe zones” proposed by Lewinnek et al [15] are really the zones lowering the risk of hip dislocation after a THA [37,38].

Knowledge of how different approaches impact stem and cup positions in a total hip arthroplasty might be crucial for achieving proper implant placement.

Even though the study by Yue et al. [18] reported differences in some radiographic results between LA and DAA, this narrative review is, to our best knowledge, the first study to sum up the radiological results when comparing the use of DAA with other approaches. However, there are some studies that underline the advantages of the DAA with regard to early rehabilitation and early postoperative outcomes [10,14]. The theory behind ‘DAA superiority’ is motivated by its potentially less invasive character since it is using the natural spaces between muscles.

On the other hand, Takada et al. [39] found that DAA is associated with a higher risk of nerve injury than the anterolateral approach (ALA), which is yet another aspect to consider when choosing the optimal approach. However, the more frequent occurrence of nerve injury in DAA patients did not result in a lesser clinical outcome, as it is the sensory lateral femoral cutaneous nerve, which is usually affected; therefore, it does not impair the motor joint function.

The potential limitation of our study is the lack of overall meta-analysis of the results from different studies. Another limitation is the focus mainly on the radiographic parameters, which are just some aspects of a successful surgery among others, such as the functional outcome, complications, and patient-reported outcome. The fact that our review included studies involving both cemented and cementless arthroplasties could impact the findings and therefore it should also be considered as a limitation.

The main strength of our study is that, to our best knowledge, it is the first to comprehensively compare DAA with other approaches in terms of the radiographic analysis of implant positioning. With the knowledge of how the choice of approach impacts the implant positioning, surgeons can alter their technique depending on the approach they choose in a particular case and achieve the desired result.

## 5. Conclusions

In conclusion, it can be admitted that according to this systematic review, which considered high-level studies, the type of approach in a total hip replacement may influence the components’ positioning during the surgery. Even though some differences in both femoral stem and cup positioning were underlined, there is still an insufficient number of randomized controlled studies analyzing the radiological parameters and comparing THRs performed using DAA and other approaches.

Some authors proposed the regular use of intraoperative fluoroscopy or robotic-assisted surgery in order to place a prosthesis properly. Perhaps this is the way to improve the outcome and standardize femoral stem and cup positioning. Implant placement is an essential step during THR surgery and making the final decision about it is the key toward achieving a satisfying outcome for both the surgeon and the patient. Surgeons must be aware that their choice of approach might impact their judgment on the angles and spaces inside the joint and thus alter the implant positioning.

## Figures and Tables

**Table 1:** PICOS strategy for study characteristics.

Participants	Patients Undergoing Total Hip Replacement for Treatment of End-Stage Primary Hip Osteoarthritis
Intervention	Total hip replacement with use of one of the surgical approaches to the hip joint (anterolateral, posterior, or direct lateral)
Comparisons	Patients undergoing total hip replacement via direct anterior approach to the hip joint
Outcomes	Radiographic analysis (femoral stem alignment, mean radiographic cup inclination, mean radiographic cup anteversion, mean radiographic cup abduction, position in Lewinnek’s safe zone)
Study design	Randomized controlled trial

**Table 2:** Participants’ characteristics in analyzed studies.

References, Year Compared Approaches	Number of Participants in Each Group (Females/Males, Left/Right Hips)	Patients’ Characteristics Age (years); BMI (kg/m^2^)	Implants Used
Nistor [8], 2017 DAA vs. LA	DAA: 35 (F/M: 26: 9, L/R: 19: 16) LA: 35 (F/M: 16: 19, L/R: 18: 17)	DAA: 67 (53.5, 72.5); 27.45 ± 3.76 LA: 64 (54.5, 67.5); 28.63 ± 3.12	Metabloc™ uncemented femoral stem system, cobalt–chrome Versys^®^ 32 mm diameter femoral head, polyethylene liner form Trilogy^®^ acetabular system, and Trilogy^®^ uncemented acetabular system shell, with acetabular self-tapping bone screws if needed (Zimmer Warsaw, IN 46,580, USA).
Reichert [21], 2018 DAA vs. LA	DAA: 73 (F/M: 32: 45, L/R: NA) LA: 50 (F/M: 32: 49, L/R: NA)	DAA 63.2 (44–77); 28.1 ± 3.7 LA: 61.9 (50–78); 28.3 ± 3.4	Trilogy or Allofit cups (Trilogy^®^ Acetabular Hip System; Allofit^®^ Acetabular Cup System), the non-cemented M/L Taper stem, or the cemented M. E. Müller straight stem. Overall, the anterior group included four cemented stems, while the lateral group included five cemented stems.
Dienstknecht [22], 2014 DAA vs. LA	DAA: 55 (F/M 33: 22, L/R: 27:28) LA: 88 (F/M 47: 41, L/R: 47:41)	DAA: 61.9 ± 12.1 (33–85); 27.6 ± 6.0 (15.7–42.0) LA: 61.3 ± 11.6 (35–89); 30.1 ± 5.6 (17.6–48.8)	Pressfit acetabular components and cement-free hydroxyapatite-coated stems with metal heads were used. Five patients in the Bauer group and one patient in the Micro-hip group received a cemented stem because of poor bone stock.
Cheng [23], 2017 DAA vs. PA	DAA: 35 (F/M: 20: 15, L/R: NA) LA: 38 (F/M: 20: 18, L/R: NA)	DAA: 59 (IQR54, 69); 27.7 (25.8, 30.0) LA: 62.5 (IQR55, 69); 28.3 (24.8, 31.1)	R3 acetabular system and Anthology femoral stem.
Taunton [24], 2014 DAA vs. PA	DAA: 27 (F/M: 15: 12, L/R: NA) LA: 27 (F/M: 14: 13, L/R: NA)	DAA: 62.05; 27.7 PA: 66.4; 29.2	The same femoral component design (Corail; DePuy, Warsaw, Indiana) and the same acetabular component design (Pinnacle; DePuy) were used in every case.
Barret [25], 2013 DAA vs. PLA	DAA: 43 (F/M: 14: 29, L/R: 21:22) PLA: 44 (F/M: 25: 19, L/R: 20:24)	DAA: 61.4 ± 9.2; 30.7 ± 5.4 PLA: 63.2 ± 7.7; 29.1 ± 5.0	Corail Total Hip System femoral stem, a Pinnacle Acetabular Cup System cup, an AltrX cross-linked polyethylene liner, and a cobalt chromium-molybdenum femoral head with size 28, 32, or 36 mm.
Zhao [26], 2017 DAA vs. PLA	DAA: 60 (F/M: 36: 24, L/R: NA) PLA: 60 (F/M: 34: 26, L/R: NA)	DAA: 64.88 ± 12.13; 25.58 ± 2.83 PLA:62.18 ± 14.72; 24.35 ± 3.10	N/A
Brun [28], 2019 DAA vs. LA	DAA: 84 (F/M: 59: 25, L/R: NA) LA: 80 (F/M: 50: 30, L/R: NA)	DAA: 67.2 ± 8.6; 27.7 ± 3.6 LA:65.6 ± 8.6; 27.6 ± 3.9	Cemented cup (Marathon; DePuy, Warsaw, IN, USA), uncemented stem (Corail; DePuy), and ceramic head with a diameter of 32 mm (BIOLOX Forte; CeramTec, Plochingen, Germany).
Taunton [27], 2018 DAA vs. PA	DAA: 52 PA: 49	DAA: 65 ± 10 (38–84); 29 ± 22 (19–39) PA: 64 ± 11 (37–85); 30 ± 4 (22–39)	Hemispherical uncemented acetabular component (Pinnacle^®^; DePuy Orthopaedics Inc, Warsaw, IN, USA), hydroxyapatite-coated femoral stem (Corail^®^; DePuy Orthopaedics Inc), and Biolox^®^ delta ceramic femoral head (CeramTec GmbH, Plochingen, Germany).

List of abbreviations: F—females, M—males, L—left, R—right, DAA—direct anterior approach, LA—direct lateral approach, PA—posterior approach, PLA—posterolateral approach, NA—not available.

**Table 3:** Cochrane Collaboration’s tool risk of bias assessment.

References	Randomization Process	Deviations from Intended Interventions	Missing Outcome Data	Measurement of the Outcome	Selection of the Reported Result	Overall Bias
Nistor [8]	+	+	+	+	+	+
Reichert [21]	+	+	+	+	+	+
Dienstknecht [22]	+	+	+	Not stated	+	+
Cheng [23]	+	+	+	-	+	+
Taunton [24]	+	+	+	-	+	+
Barret [25]	+	+	+	-	+	+
Zhao [26]	+	+	+	+	+	+
Taunton [27]	+	+	+	-	+	+
Brun [28]	+	+	+	+	+	+

“+” denotes low risk of bias, “-” denotes high risk of bias.

**Table 4:** Femoral stem alignment in analyzed studies.

References, Year Compared Approaches	DAA	LA	PA	PLA
Nistor [8], 2017 DAA vs. LA	1.40° (SD 0.99°) varus	1.29° (SD 1.13°) varus		
Reichert [21], 2018 DAA vs. LA	5.5% varus 2% valgus 92.5% neutral	4% varus 2% valgus 94% neutral		
Dienstknecht [22], 2014 DAA vs. LA	2.6° (SD 2.1°) varus	2.8° (SD 2.2°) varus		
Cheng [23], 2017 DAA vs. PA	1.09° varus	1.62° varus		
Barret [25], 2013 DAA vs. PLA	2% varus 0% valgus 98% neutral			26% varus 0% valgus 74% neutral
Taunton [24], 2014 DAA vs. PA	4 varus/52 operated		6 varus, 2 valgus/49 operated	
Brun [28],2019 DAA vs. LA	3.1° (SD 1.5°) varus	2.9° (SD 1.1°) varus		

**Table 5:** Mean radiographic cup inclination angles in analyzed studies.

References, Year Compared Approaches	DAA	LA	PA	PLA
Nistor [8], 2017 DAA vs. LA	36.97° (SD 1.85°)	39.63° (SD 2.88°)		
Reichert [21], 2018 DAA vs. LA	38.6° (SD 5.7°)	40.3° (SD 6.2°)		
Dienstknecht [22], 2014 DAA vs. LA	48.1° (SD 6.0°)	49.7° (SD 6.0°)		
Cheng [23], 2017 DAA vs. PA	46.07°		45.86°	
Taunton [24], 2014 DAA vs. PA	38.0°		40.0°	
Barret [25], 2013 DAA vs. PLA	47.1° (SD 6.1°)			42.4° (SD 7.6°)
Zhao [26], 2017 DAA vs. PLA	41.3°			40.8°
Taunton [27], 2018 DAA vs. PA	37° (SD 5°)		39° (SD 6°)	
Brun [28],2019 DAA vs. LA	49.5° (SD 7.4°)	47.0° (SD 6.0°)		

**Table 6:** Mean radiographic cup anteversions in analyzed studies.

References, Year Compared Approaches	DAA	LA	PA	PLA
Cheng [23], 2017 DAA vs. PA	24.57°		20.34°	
Taunton [24], 2014 DAA vs. PA	26°		29°	
Barret [25], 2013 DAA vs. PLA	20.1° (SD 5.9°)			25.8° (SD 8.1°)
Zhao [26], 2017 DAA vs. PLA	17.1°			21.3°
Taunton [27], 2018 DAA vs. PA	23° (SD 4°)		25° (SD 6°)	
Brun [28], 2019 DAA vs. LA	9.4° (SD 4.8°)	5.8° (SD 4.3°)		
